# Gender difference in association between low muscle mass and risk of non-alcoholic fatty liver disease among Chinese adults with visceral obesity

**DOI:** 10.3389/fnut.2023.1026054

**Published:** 2023-01-13

**Authors:** Yayun Lu, Qing Xia, Liangyu Wu, Zhiping Xie

**Affiliations:** Health Examination Center, Huadong Sanatorium, Wuxi, China

**Keywords:** non-alcoholic fatty liver disease, visceral obesity, gender, age, low muscle mass

## Abstract

**Background and aims:**

Although the association between low muscle mass and the risk of non-alcoholic fatty liver disease is well-known, it has not been explored in viscerally obese populations by gender. Besides, whether low muscle mass still increases the NAFLD risk in subjects with visceral obesity, independent of obesity, is still unknown. The aim of this study was to explore the gender-specific association between low muscle mass and the risk of non-alcoholic fatty liver disease (NAFLD) in subjects with visceral obesity.

**Methods:**

Overall, 1,114 participants aged 19–89 years were recruited in this retrospective study. Liver disease was diagnosed by hepatic ultrasound. Skeletal muscle mass was estimated by bioimpedance analysis and defined by the appendicular skeletal muscle index (ASMI). Gender-specific differences in the ASMI value were compared between NAFLD and control groups. Restricted cubic spline and multivariate logistic regression were performed to analyze the association (stratified by gender and age) between the ASMI and the risk of NAFLD, respectively.

**Results:**

Middle-aged females (40–60 years) and males (of any age) with NAFLD had a significantly lower ASMI compared with controls (*P*-value < 0.05). An inverse linear association was found between the ASMI and risk of NAFLD (all *P*_fornon−linearity_ > 0.05). Lower quartiles of the ASMI conferred independent risk of NAFLD compared to higher quartiles (all *P* for trend < 0.001). Low muscle mass conferred a higher risk of NAFLD in middle-aged females (adjusted odds ratio = 2.43, 95% confidence interval: 1.19–4.95) and males [18–39 years: 3.76 (1.79–7.91); 40–60 years: 4.50 (2.16–9.39); and >60 years: 4.10 (1.13–14.84)]. Besides, Low muscle mass and low muscle mass with obesity increase the risk of developing NAFLD, independent of obesity.

**Conclusion:**

Among those with visceral obesity, low muscle mass increased the risk of NAFLD in males of any age, and middle-aged females, this may be explained by the postmenopausal decline in estrogen.

## Introduction

Non-alcoholic fatty liver disease (NAFLD) is characterized by abnormal lipid accumulation in the liver without long-term alcohol consumption and is currently the main cause of chronic liver disease. The condition encompasses simple steatosis, non-alcoholic steatohepatitis, advanced fibrosis, and end-stage liver disease, which is related to cirrhosis and hepatocellular carcinoma (1–4). The prevalence of NAFLD ranges from 25 to 30% worldwide; (1) however, it is as high as 36% in China (5, 6). NAFLD has been proven to be positively associated with systemic diseases and burdens the government health services (7). Individuals with insulin resistance, dyslipidemia, or visceral obesity are commonly susceptible to NAFLD (8). As excessive loss of skeletal muscle promotes insulin resistance and exacerbates energy expenditure (9), low muscle mass is independently associated with metabolic abnormalities. Several studies have explored the adverse effects of low skeletal muscle mass in terms of NAFLD risk (10). A perspective cohort study found that less skeletal muscle was consistently associated with a significantly increased risk of NAFLD over 12 years follow-up (11). Another meta-analysis including five cross-sectional studies on 27,804 participants, demonstrated an ~1.5-fold increase in the risk of NAFLD among subjects with sarcopenia (12).

Insulin resistance or dyslipidemia are commonly associated with the development of obesity and low muscle mass (13, 14). Few studies have explored the effect of low muscle mass on NAFLD risk, independent of obesity effect. In this context, gender related difference in the prevalence of NAFLD vary in contemporary literature. Males are more likely to develop NAFLD compared to females due to a higher total lean mass loss (15). Middle-aged males demonstrate a higher prevalence of NAFLD, whereas females show a steadily increasing prevalence with age (16, 17). To our knowledge, the predominant obesity phenotype in the Asian population is abdominal obesity, which is characterized by an excess of visceral adipose tissue deposition (18). Furthermore, excessive visceral adipose is significantly associated with fatty liver disease, it remains unknown whether low muscle mass still increases the NAFLD risk in subjects with visceral obesity, independent of obesity. This study aimed to evaluate the gender-specific impact of low muscle mass on NAFLD risk in Chinese adults with visceral obesity using a health check-up database.

## Methods

### Study participants

This retrospective study randomly enrolled 1,380 participants with visceral obesity, who had initially attended the annual health examination that was conducted at the Huadong Sanatorium between June 2020 and May 2021. Participants who had undergone hepatic ultrasonography and completed the survey for demographic, personal life style, medical history, and biochemical and anthropometric data were selected. Exclusion criteria: (1) alcohol consumption ≥3 times per week at least 12 months; (2) history of viral hepatitis; (3) receiving antidiabetic, anti-hypertension or lipid-lowering therapy currently. (4) Incomplete personal and medical information.

A total of 1,114 participants including 626 females and 488 males (aged 19–89 years) were finally enrolled. The mean age (±standard deviation) of the subjects was 48.46 ± 13.85 years. The demographic data included age, gender, cigarette use; smoking was defined as the smoking of at least three cigarettes per day in a year. This retrospective study was conducted in accordance with the principles of the Declaration of Helsinki and was approved by the Ethics and Research Committee of the Huadong Sanatorium Health Examination Center (approval number. ECHS2022-11). Personal information was anonymized to protect patients' privacy; statistical analyses were conducted with strict confidentiality and were only used for scientific purposes. The requirement for informed consent was therefore waived.

### Body composition and anthropometric measurements

For all participants, assessment of the body composition was performed using the segmental multifrequency bioelectrical impedance analysis system (InBody 4.0, InBody Co., South Korea). A visceral fat area of ≥100 cm^2^ was defined as visceral obesity. The following validated equitation derived by Janssen et al. was used to evaluate the skeletal muscle mass in each subject (19): skeletal muscle mass (kilograms) = [(height^2^/BIA resistance × 0.401) + (gender × 3.825) + (age × −0.071)] +5.102, where height is recorded in centimeters; BIA resistance is recorded in ohms; For gender, men is coded as 1, and 0 for women; age is in years. The appendicular skeletal mass (ASM) was the calculated sum of lean muscle mass in bilateral upper and lower limbs. The appendicular skeletal mass index (ASMI) was calculated by dividing the ASM by the square of the body height (kg/m^2^). As recommended by the Asian Working Group for Sarcopenia (AWGS), low muscle mass was defined by an ASMI of ≤ 7.0 kg/m^2^ for males and ≤ 5.7 kg/m^2^ for females (20). Body weight, height, and waist circumference (WC) were measured by professional trained nurses. The body mass index (BMI) was calculated by following formula: body weight (kg)/height (m)^2^; the WC was measured to the nearest 0.1 cm at the level of the highest point of the iliac crest (21).

**Figure 1:**
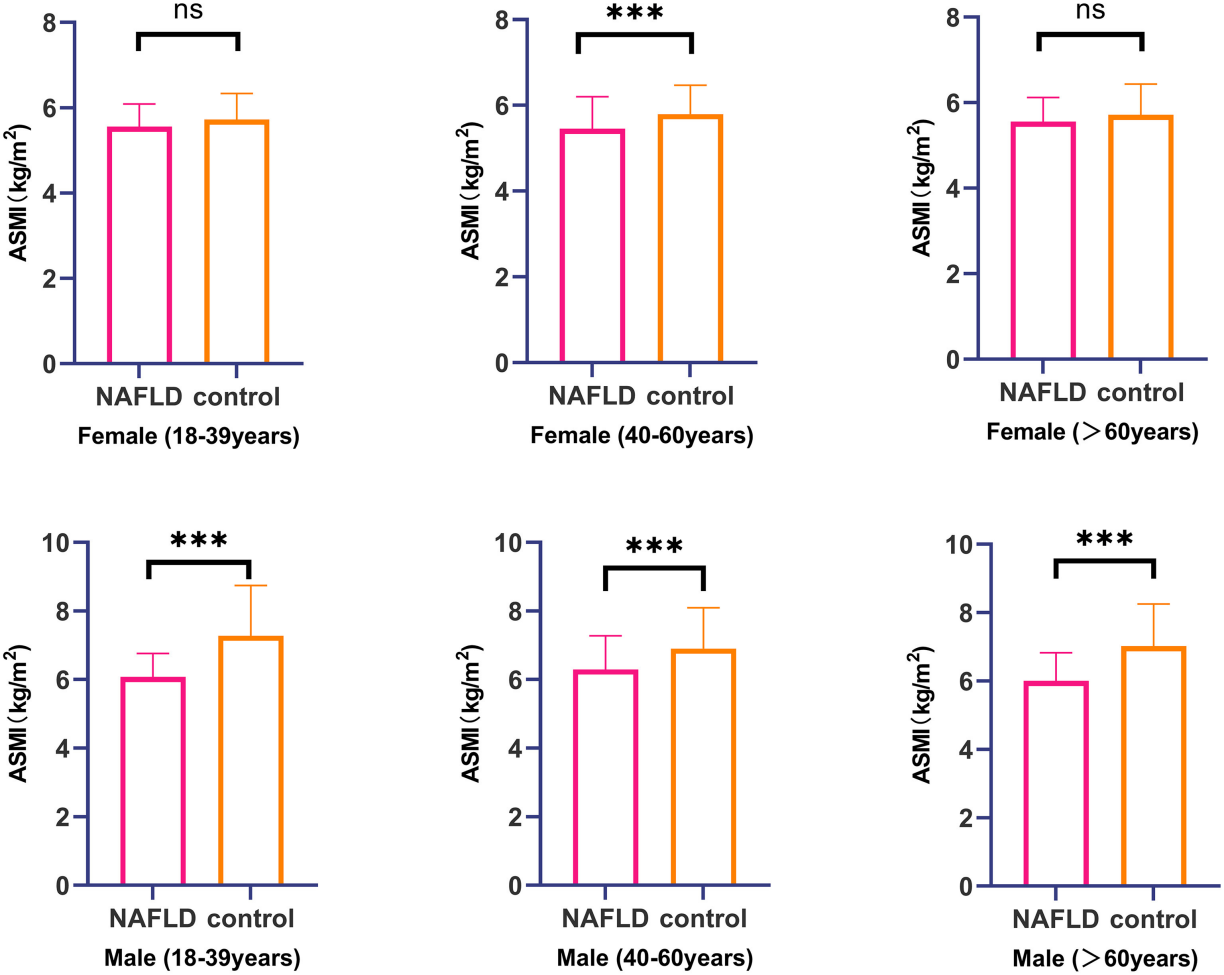
Comparison of ASMI value between NAFLD group and control group stratified by gender and age range. ***, *P* < 0.001; NS, no significant.

We also compared the ASMI value by age between the NAFLD and control groups (Figure 1). Only middle-aged females with NAFLD had significantly lower ASMI values than the controls (*P*-value < 0.05); however, the ASMI values of males with NAFLD were significantly lower than those of the controls, irrespective of age (*P*-value < 0.001).

### Laboratory and clinical measurements

Blood specimens (8–10 ml) were collected from the antecubital vein after a 12 h overnight fast. The serum was placed at room temperature for 30 min and centrifuged at 3,000 rpm for 10 min. The parameters evaluated included fasting plasma glucose (FPG), triglycerides (TGs), total cholesterol (TC), low-density-lipoprotein cholesterol (LDL-C), high-density-lipoprotein cholesterol (HDL-C), serum aspartate aminotransferase (AST), alanine aminotransferase (ALT), white blood cells (WBC), neutrophils (NE), and lymphocytes (LY). All blood samples were tested within 24 h at the medical laboratory center of the Huadong Sanatorium.

Diabetes mellitus was defined by a FPG level of ≥126 mg/dl or current intake of antidiabetic medication, while hypertension was defined by a systolic blood pressure of ≥140 mmHg, diastolic blood pressure of ≥90 mmHg, or current intake of anti-hypertensive treatment.

### Assessment of NAFLD

NAFLD was measured using high-resolution abdominal ultrasonography (SIEMENS ACUSON S2000 ABVS) after 12 h of fasting. Trained radiologists performed the ultrasonography and diagnosed NAFLD based on the presence of hepatic steatosis, following exclusion of long-term alcohol consumption and viral or autoimmune liver disease. The diagnosis was made based on the following characteristics on the abdominal ultrasound images (22): diffuse increase in the near field echo of the liver (“bright liver”) and greater echogenicity of the liver than of the kidney or spleen; vascular blurring and poor visibility of the posterior right lobe due to deep attenuation.

### Statistical analysis

All statistical analyses were performed using SPSS 25.0 and STATA 16.0 software packages. Continuous variables are reported as means ± standard deviation or medians with interquartile ranges (based on evaluation of normal distribution by the Shapiro–Wilk test). Categorical variables are presented as numbers (*n*) with percentages (%). Baseline characteristics and laboratory parameters (specified by gender) were compared using the Student's *t*- or Mann–Whitney *U*-tests for continuous variables and the chi-square test for categorical variables. Restricted cubic spline regression was used to estimate the gender-specific association between the ASMI and NAFLD in a fully adjusted model. The knots were placed at the 5th, 35th, 65th, and 95th percentiles. Multivariable logistic regression was also performed (after adjusting for potential confounders) to determine the relationship between ASMI quartiles (using gender-specific quartiles) and NAFLD, stratified by gender; the fourth quartile was used as the reference group. Model 1 was adjusted for age, smoking, diabetes, hypertension, WC, and BMI. Model 2 was adjusted for model 1 plus TG, LDL-c, ALT, and AST. Adjusted odds ratios (ORs) have been presented with 95% confidence intervals (CIs). The *P* for trend was evaluated for the linear trend test using the median value of ASMI as a continuous variable in the adjusted models. We also performed subgroup analysis based on gender in different age ranges; the *P* for interaction was further explored using the Wald test. A two-tailed *P*-value of < 0.05 was considered statistically significant.

## Results

### Baseline characteristics of the study population

The basic demographic and laboratory characteristics (stratified by gender) of the 1,114 participants have been presented in Table 1. The prevalence of NAFLD among females and males was 35.9% (*n* = 225) and 51.4% (*n* = 251), respectively. In those with NAFLD, the prevalence of low muscle mass was statistically higher in males than in females (84.1 vs. 23.1%, χ^2^ = 178.28, *P* < 0.001). In addition, males and females with NAFLD had lower ASMI values than the control group (all *P-*values < 0.001). Participants with NAFLD were older, and a higher proportion were smokers and drinkers compared to the control group (all *P-*values < 0.05). The values of the BMI, WC, systolic blood pressure, diastolic blood pressure, and levels of FPG, TG, LDL-C, ALT, and AST were also significantly higher in subjects with NAFLD (all *P-*values < 0.05).

**Table 1:** Descriptive characteristics of participants with and without NAFLD, by gender (*n* = 1,114).

**Characteristics**	**Overall (*n* = 1,114)**	**Female (*****n*** = **626)**		***P*-value**	**Male (*****n*** = **488)**		***P*-value**
		**Control (*****n*** = **401)**	**NAFLD (*****n*** = **225)**		**Control (*****n*** = **237)**	**NAFLD (*****n*** = **251)**	
Age (years)	48.46 ± 13.85	47.08 ± 13.38	53.80 ± 12.53	<0.001	45.65 ± 13.42	48.54 ± 14.85	0.025
Smoking (*n*, %)	229 (20.6)	19 (4.7)	14 (6.2)	0.458	76 (32.1)	120 (47.8)	<0.001
WC (cm)	87.81 ± 9.86	79.52 ± 5.79	85.81 ± 7.01	<0.001	94.27 ± 6.85	96.75 ± 7.58	<0.001
BMI (kg/m^2^)	27.11 ± 3.44	24.77 ± 2.33	27.32 ± 3.36	<0.001	28.47 ± 2.78	29.36 ± 3.32	0.001
SBP (mmHg)	124.48 ± 16.32	117.23 ± 15.75	129.02 ± 16.37	<0.001	125.52 ± 12.83	131.03 ± 15.67	<0.001
DBP (mmHg)	75.46 ± 10.34	71.79 ± 9.24	77.69 ± 10.33	<0.001	75.17 ± 9.78	79.61 ± 10.50	<0.001
Hypertension (*n*, %)	172 (15.4)	30 (7.5)	57 (25.3)	<0.001	28 (11.8)	57 (22.7)	0.002
FPG (mmol/L)	5.74 ± 1.25	5.59 ± 1.08	5.95 ± 1.27	<0.001	5.62 ± 1.20	5.91 ± 1.48	0.015
Diabetes (*n*, %)	111 (10.0)	22 (5.5)	34 (15.1)	<0.001	18 (7.6)	37 (14.7)	0.015
TC (mmol/L)	5.01 [4.43, 5.58]	5.01 [4.43, 5.53]	5.01 [4.41, 5.77]	0.291	5.00 [4.39, 5.61]	5.02 [4.47, 5.54]	0.930
TG (mmol/L)	1.62 [1.11, 2.52]	1.45 [1.02, 2.18]	2.14 [1.32, 3.06]	<0.001	1.49 [1.05, 2.20]	1.73 [1.15, 2.86]	0.007
LDL-C (mmol/L)	3.16 [2.70, 3.69]	3.11 [2.61, 3.65]	3.20 [2.83, 3.82]	0.017	3.16 [2.69, 3.55]	3.24 [2.79, 3.97]	0.018
HDL-C (mmol/L)	1.25 [1.06, 1.46]	1.28 [1.08, 1.50]	1.26 [1.09, 1.42]	0.425	1.23 [1.03, 1.42]	1.22 [1.06, 1.45]	0.446
ALT (IU/L)	23.60 [16.38, 36.75]	20.50 [14.90, 30.05]	25.10 [18.20, 36.45]	<0.001	23.00 [16.20, 35.00]	28.20 [17.40, 40.60]	0.007
AST (IU/L)	22.45 [18.40, 28.80]	20.00 [17.00, 24.30]	24.30 [20.25, 28.05]	<0.001	21.80 [18.10, 28.15]	27.80 [20.30, 34.60]	<0.001
WBC (× 10^9^/L)	6.18 [5.32, 7.30]	6.27 [5.31, 7.30]	6.20 [5.33, 7.28]	0.876	6.09 [5.19, 7.28]	6.10 [5.44, 7.29]	0.251
NE (× 10^9^/L)	3.38 [2.73, 4.10]	3.47 [2.72, 4.08]	3.42 [2.74, 4.15]	0.925	3.33 [2.76, 4.04]	3.31 [2.71, 4.17]	0.772
LY (× 10^9^/L)	2.23 [1.89, 2.65]	2.27 [1.88, 2.70]	2.25 [1.95, 2.70]	0.713	2.10 [1.83, 2.65]	2.22 [1.90, 2.68]	0.162
ASMI (kg/m^2^)	6.12 ± 1.05	5.77 ± 0.65	5.50 ± 0.67	<0.001	7.04 ± 1.29	6.33 ± 0.92	<0.001
Low muscle mass (*n*, %)	459 (41.2)	55 (13.6)	50 (22.4)	0.003	134 (56.5)	211 (84.1)	<0.001

Categorical variables have been presented as n (%); continuous variables have been presented as mean ± standard deviation or median (interquartile range).

WC, waist circumference; BMI, body mass index; SBP, systolic blood pressure; DBP, diastolic blood pressure; FPG, fasting plasma glucose; TG, triglycerides; TC, total cholesterol; LDL-C, low-density-lipoprotein cholesterol; HDL-C, high-density-lipoprotein cholesterol; AST, serum aspartate aminotransferase; ALT, alanine aminotransferase; WBC, white blood cells; NE, neutrophils; LY, lymphocytes; ASMI, appendicular skeletal mass index.

### Dose-response relationship between ASMI values and NAFLD risk in male and female subjects

**Figure 2:**
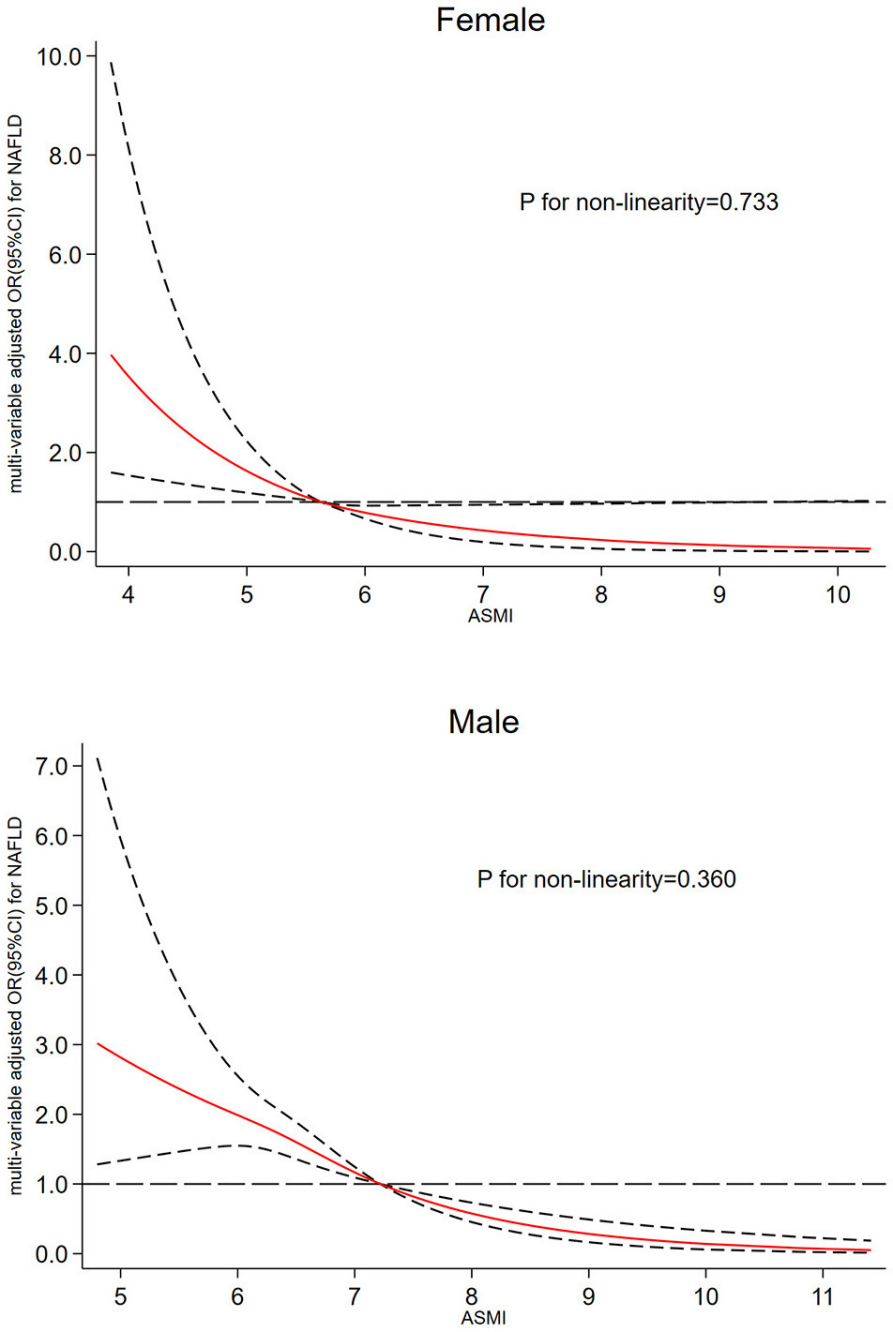
Dose-response relationship between ASMI and risk of NAFLD in female and male. The restricted cubic spline regression analysis was adjusted for age, smoking, diabetes, hypertension, WC, BMI, TG, LDL-c, ALT, and AST. The long dashed line represents OR is equal to 1, red line and the area between the short dashed lines means ORs and their 95% CI.

The gender-specific dose-response relationship between ASMI values and NAFLD risk has been demonstrated in Figure 2. The horizontal line represents the 5th, 35th, 65th, and 95th percentiles of ASMI, restricted cubic spline regression line (red line) represents multivariable adjusted ORs for NAFLD with four knots located at 5th, 35th, 65th, and 95th percentiles of ASMI, and multivariable adjusted restricted cubic spline analysis demonstrated that decreasing value of ASMI significantly increased the risk of developing NAFLD when ASMI values was below 5.63 kg/m^2^ and 7.21 kg/m^2^ in females and males respectively, but showed a significant protective effect above this value. Besides, a linear relationship between ASMI and NAFLD was detected in both females (*P*_fornon − linearity_ = 0.733) and males (*P*_fornon − linearity_ = 0.360).

### Association between each quartile of ASMI and risk of NAFLD by gender

Table 2 presents the results of multivariable logistic regression for the association between ASMI values as continuous and categorical variables with the risk of NAFLD in females and males. In all the regression models, adjusted for age, smoking, diabetes, hypertension, WC, and BMI were adjusted in model 1. Model 2 was further adjusted for TG, LDL-c, ALT, and AST. Per standard deviation increase in ASMI value was significantly associated with a lower prevalence of NAFLD in both females (adjusted OR = 0.62, 95% CI: 0.49–0.77) and males (adjusted OR = 0.51, 95% CI: 0.40–0.63). Besides, this study also confirmed that decreasing quartiles of ASMI values significantly increased the risk of NAFLD in males (*P*_fortrend_ < 0.001). The adverse impact of ASMI on NAFLD was statistically significant in females (OR_Q1vs.Q4_ = 2.81, 95% CI = 1.57–5.02) and males (OR_Q1vs.Q4_ = 4.92, 95% CI = 2.74–8.84).

**Table 2:** Gender-specific association between each quartile of ASMI values and risk of NAFLD by gender.

**Variables**	**Total (*n*, %)**	**NAFLD (*n*, %)**	**Crude model**	**Model 1**	**Model 2**
Female	626	225 (35.9)			
Per SD increase			0.63 (0.52–0.76)	0.61 (0.49–0.76)	0.62 (0.49–0.77)
Q1	88 (14.1)	42 (47.7)	3.08 (1.87–5.06)	2.95 (1.66–5.24)	2.81 (1.57–5.02)
Q2	145 (23.2)	44 (30.3)	3.28 (1.99–5.39)	3.07 (1.73–5.45)	2.89 (1.62–5.17)
Q3	216 (34.5)	75 (34.7)	1.55 (0.92–2.59)	1.43 (0.79–2.60)	1.46 (0.80–2.66)
Q4	177 (28.3)	64 (36.2)	1.00 (reference)	1.00 (reference)	1.00 (reference)
*P* for trend			<0.001	<0.001	<0.001
Male	488	251 (51.4)			
Per SD increase			0.51 (0.42–0.63)	0.50 (0.41–0.64)	0.51 (0.40–0.63)
Q1	190 (38.9)	126 (66.3)	4.93 (2.86–8.50)	4.80 (2.73–8.44)	4.92 (2.74–8.84)
Q2	135 (27.7)	73 (54.1)	3.86 (2.26–6.59)	3.43 (1.96–6.01)	3.58 (2.00–6.41)
Q3	62 (12.7)	24 (38.7)	2.86 (1.68–4.86)	2.69 (1.55–4.68)	2.93 (1.65–5.20)
Q4	101 (20.7)	28 (27.7)	1.00 (reference)	1.00 (reference)	1.00 (reference)
*P* for trend			<0.001	<0.001	<0.001

Model 1: adjusted for age, smoking, diabetes, hypertension, WC, and BMI.

Model 2: adjusted for age, smoking, diabetes, hypertension, WC, BMI, TG, LDL-c, ALT, and AST.

### Association between low muscle mass and NAFLD risk stratified by gender and age

**Figure 3:**
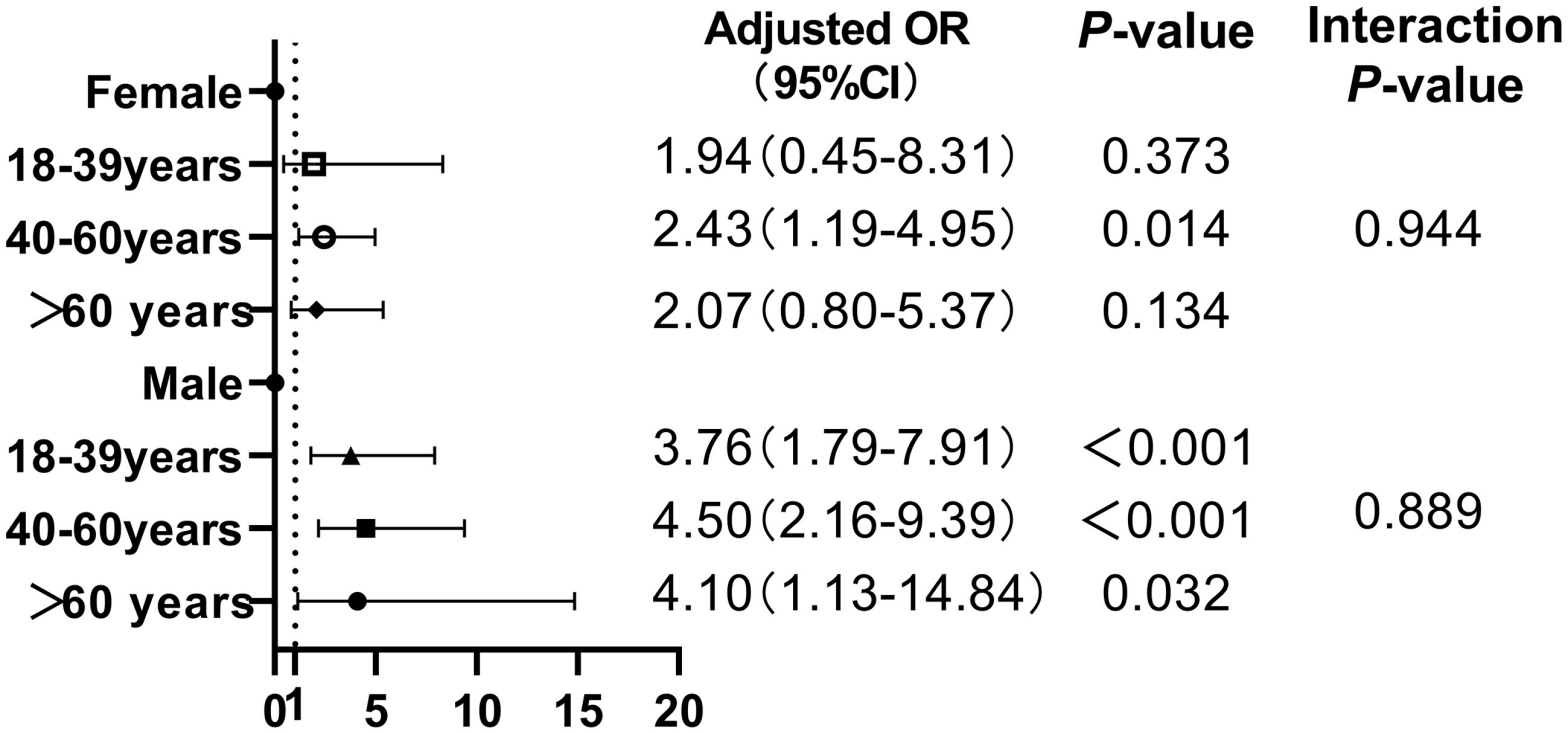
Association between low muscle mass and NAFLD risk stratified by gender and age.

As shown in Figure 3, after adjusting for potential confounding factors including smoking, diabetes, hypertension, WC, BMI, TG, LDL-c, ALT, and AST, only middle-aged females (40–60 years) with low muscle mass had a significant risk of NAFLD (adjusted OR = 2.43, 95% CI = 1.19–4.95, *P* = 0.014); in contrast, low muscle mass increased the risk of NAFLD in males of any age, the ORs (95% CI) for NAFLD in males with low muscle mass were 3.76 (1.79–7.91) for those aged 18–39 years, 4.50 (2.16–9.39) for those aged 40–60 years and 4.10 (1.13–14.84) for those over 60 years. Additionally, there were no significant interactions between low muscle mass and age on NAFLD risk (all *P*_forinteraction_ > 0.05).

### Association between obesity, low muscle mass and low muscle mass with obesity on risk of NAFLD by gender

Table 3 presents the results of association between obesity, low muscle mass and low muscle mass with obesity on risk of NAFLD in female and male subjects after adjusting for confounding factors. Low muscle mass significantly increased the risk of NAFLD in both females (adjusted OR = 1.82) and males (adjusted OR = 4.60), independent of obesity (all *P-*value < 0.001). Moreover, Participants with both low muscle mass and obesity had the highest odds ratio of developing NAFLD (adjusted OR =10.60 for females and 4.70 for males).

**Table 3:** Gender-specific association between obesity, low muscle mass and obesity with low muscle mass on risk of NAFLD by gender.

**Variables**	**Total (*n*, %)**	**NAFLD (*n*, %)**	**Crude model**	**Model 1**	**Model 2**
Female	626	225 (35.9)			
Control	422	107 (25.4)	1.00 (reference)	1.00 (reference)	1.00 (reference)
Obesity	99	66 (66.7)	5.90 (3.67–9.44)	0.82 (0.36–1.88)	0.91 (0.39–2.10)
Low muscle mass	89	35 (39.3)	1.91 (1.18–3.08)	1.83 (1.08–3.12)	1.82 (1.06–3.14)
Obesity + low muscle mass	16	15 (93.8)	44.16 (5.76–338.28)	10.25 (1.14–91.85)	10.60 (1.18–95.42)
Male	488	251 (51.4)			
Control	62	13 (21.0)	1.00 (reference)	1.00 (reference)	1.00 (reference)
Obesity	76	26 (34.2)	1.96 (0.90–4.25)	1.33 (0.53–3.31)	1.31 (0.51–3.33)
Low muscle mass	148	80 (54.1)	4.43 (2.22–8.86)	3.98 (1.96–8.11)	4.60 (2.18–9.68)
Obesity + low muscle mass	202	132 (65.3)	7.11 (3.61–13.983)	4.91 (2.21–10.92)	4.70 (2.06–10.74)

Model 1: adjusted for age, smoking, diabetes, hypertension, WC, and BMI.

Model 2: adjusted for age, smoking, diabetes, hypertension, WC, BMI, TG, LDL-c, ALT, and AST.

## Discussion

This was the first study to investigate the gender- and age-specific association between low muscle mass and the risk of NAFLD in Chinese adults with visceral obesity. Our study confirmed that ASMI values were significantly lower in males and middle-aged females with NAFLD than in those without NAFLD. In addition, ASMI values showed an inverse relationship with the risk of NAFLD in both males and females. After adjusting for confounding factors, multivariable logistic regression analysis demonstrated that in the subjects with visceral obesity, low muscle mass was associated with a higher risk of development of NAFLD in middle-aged females and males of any age. Besides, Low muscle mass with obesity or low muscle mass alone increase the risk of developing NAFLD, independent of obesity.

Previous studies have demonstrated that visceral adiposity increases the risk and severity of NAFLD (23–25). However, studies have not evaluated whether low muscle mass remains an independent contributor to NAFLD in populations with visceral obesity. As an endocrine organ, visceral adipose tissue (VAT) has been reported to be biologically active; excessive VAT contributes to considerable insulin resistance, expresses larger numbers of androgen receptors, and releases higher levels of adiponectin and IL-6 than subcutaneous adipose tissue (26). An excess of activated adipocytes in VAT could induce the accumulation of pro-inflammatory macrophages and dysregulated immune cells, which produce various adipokines, immune cell-released cytokines, and chemokines (27, 28). Therefore, it is necessary to investigate the impact of low muscle mass on the risk of NAFLD in populations with visceral obesity. Our study demonstrated that low muscle mass remains a statistically significant risk factor for NAFLD in both males and middle-aged females with visceral obesity. Besides, a recent study indicated that subjects with excess VAT had high risk for metabolic syndrome components and the development of hepatic steatosis, and VAT rather than subcutaneous abdominal fat is more effective than BMI in predicting the presence of NAFLD. Thus, reducing abdominal fat excess is the key therapeutical approach of NAFLD during the earliest stage of its development (29).

Middle-aged females (40–60 years) with low muscle mass were more susceptible to develop NAFLD compared with other age groups (OR = 2.43, 95% CI: 1.19–4.95). A study has shown the prevalence of NAFLD to be significantly higher in postmenopausal females compared their premenopausal counterparts (30). Circulating levels of estrogen may therefore be considered as a protective biomarker against the development of hepatic steatosis. In support of this hypothesis, a cohort study in patients with NAFLD reported a higher risk of severe fibrosis in males than in premenopausal females; however, the risk levels were similar between males and postmenopausal females. The findings indicate a protective effect of estrogen against liver fibrosis in NAFLD (31, 32). A reduction of estrogen expression could increase the accumulation of insulin resistance and visceral adiposity (33). In this context, physiologic concentrations of estrogen inhibit the secretion of proinflammatory cytokines and reduce the risk of developing NAFLD (34). A study confirmed that hormone replacement therapy improves hepatic function and reduces liver enzymes in postmenopausal females (30). Testosterone acts as a potent anabolic hormone and promotes the synthesis of muscle proteins; in this context, a recent study found that lower serum levels of total testosterone are an important contributor to the risk of NAFLD in males, independent of metabolic risk factors and obesity (35, 36). Our study also demonstrated that middle-aged and older adults with visceral obesity were at higher of developing NAFLD compared with younger individuals. Based on these findings, it may be assumed that sex hormones could be responsible for sex-specific differences in the prevalence of NAFLD and the association between sarcopenia and the risk of NAFLD.

The pathophysiologic mechanism linking sarcopenia or low muscle mass and the risk of NAFLD warrants further investigation. Previous epidemiological studies have observed insulin resistance to be a major contributor to the development of sarcopenia (37). Our study also indicated that subjects with NAFLD had a higher prevalence of T2DM compared with the control group (all *P*-values < 0.05). Sarcopenia has been described as a new diabetes complication in the elderly patients, and is characterized by loss of skeletal muscle mass combined with the decline of muscle power and function in T2DM. Impaired insulin signaling by inflammatory cytokines increases the level of plasma free fatty acids and prompts lipid deposition in the skeletal muscle and liver (38). Insulin resistance simultaneously increases the stimulation for protein degradation and eventually induces persistent muscle loss. Sarcopenia aggravates insulin resistance, as muscle is a primary tissue for peripheral insulin-mediated glucose uptake. Furthermore, chronic hyperglycemia promotes the accumulation of advanced glycosylation end products (AGEs) in skeletal muscle, and reduction of skeletal muscle performance, such as grip strength or walking speed, had been reported to be associated with AGEs (39). Besides, presence of microvascular and macrovascular complications also contributes to the high prevalence of sarcopenia in T2DM individuals, chronic kidney disease affects muscle mass, while peripheral diabetic neuropathy impairs its skeletal muscle performance, and leads to the unstable postural balance or impaired vision. Moreover, peripheral vascular disease may induce muscle ischemia and worsen muscle protein synthesis, and results in decreased skeletal muscle function (39). Therefore, individuals with T2DM deserves more attention by its high risk of developing sarcopenia. Additionally, Growth hormone, cytokines, chronic inflammation, vitamin D deficiency, and low-frequency physical activity also play an important role in sarcopenia and NAFLD development (40–42). Lifestyle changes may therefore prevent or improve sarcopenia or skeletal muscle mass and decrease the risk of NAFLD.

Lifestyle interventions, comprising enhanced physical exercise and nutritional supplementation, have been confirmed to be beneficial for patients with sarcopenia and NAFLD. Evidence had proved that efforts to prevent or relieve NAFLD should focus on reducing VAT stores and increasing muscle mass. Moderate-to-vigorous exercise for at least 30 min reduces the abdominal adipose tissue and increases muscle mass (43). In terms of the effect of exercise type on muscle regeneration, resistance training has been found to be superior to aerobic physical activity in improving knee extensor strength and handgrip in sarcopenic patients (44). In addition, exercise combined with calorie restriction can help reduce fat mass in sarcopenic and populations with visceral obesity (45). Dietary protein supplementation has also been reported to reduce sarcopenia, as it is the substrate for muscle protein synthesis. Leucine-rich whey protein and vitamin D also improve muscle mass and leg muscle (46). In summary, lifestyle changes, mainly focused on exercise and diet changes, are currently recommended for improving sarcopenia or low muscle mass and NAFLD in clinical practice.

This study has several limitations. Firstly, it had a retrospective design and could not directly prove causality. Secondly, the diagnosis of NAFLD is mainly based on hepatic ultrasound examination and cannot be confirmed histologically by liver biopsy. Finally, evaluation of muscle strength and physical performance is a prerequisite for assessing skeletal muscle function. Tests such as the short physical performance battery, usual gait speed walk test, stair climb power test, and timed-up and-go test need to be performed in addition to the calculation of ASMI values to accurately define the extent of sarcopenia. Combined evaluation is of more value than any one test alone, and low muscle mass alone may not represent the sarcopenia in most populations.

## Conclusion

In our study, low muscle mass continued to be associated with the risk of NAFLD in males with visceral obesity, irrespective of age. However, it was only associated with a significant risk of NAFLD in middle-aged females with visceral obesity. This may be explained by the postmenopausal decline in estrogen levels.

## Data availability statement

The raw data supporting the conclusions of this article will be made available by the authors, without undue reservation.

## Ethics statement

The studies involving human participants were reviewed and approved by the Ethics and Research Committee of the Huadong Sanatorium Health Examination Center. The Ethics Committee waived the requirement of written informed consent for participation.

## Author contributions

LW and ZX designed the study and critically reviewed the original manuscript. QX collected the laboratory and demographic data. YL contributed to statistical analyses, project administration, funding acquisition, and drafted the original manuscript. All authors have read and agreed to the final version of the manuscript.
